# Management of Syndesmotic Injuries in Children and Adolescents: Results of a Cross-Sectional Survey of Pediatric Orthopaedic Surgeons

**DOI:** 10.5435/JAAOSGlobal-D-24-00128

**Published:** 2024-08-15

**Authors:** Caroline E. Williams, Blair Stewig, Sang Won Lee, Benjamin J. Shore, Collin J. May

**Affiliations:** From the Department of Orthopedic Surgery, Boston Children's Hospital, Boston, MA (Dr. Williams, Ms. Stewig, Mr. Lee, Dr. Shore, and Dr. May); the Harvard Medical School, Boston, MA (Mr. Lee, Dr. Shore, and Dr. May)

## Abstract

**Introduction:**

Although syndesmotic injury management in adults has shown improved outcomes with suture-button versus screw fixation, this has not been demonstrated in pediatric populations. This study investigates trends in syndesmotic injury management by conducting a survey of pediatric orthopaedic surgeons.

**Methods:**

The Children's Orthopaedic Trauma and Infection Consortium for Evidence-Based Studies group was surveyed for information regarding their surgical fixation preference for syndesmotic ankle injuries.

**Results:**

A survey response of 100% (30/30 members) was obtained. Most of the respondents practiced in a metropolitan setting (86.7%) and reported working in a pediatric specialty hospital (73.3%). 86.7% (n = 26) treated 0 to 5, 10% (n = 3) treated 6 to 10, and 3.33% (n = 1) treated over 10 pediatric syndesmotic ankle injuries in the past year. 70% (n = 21) of respondents preferred suture-button fixation while 30% (n = 9) preferred screw fixation. Furthermore, 50% (n = 15) of respondents reported a change in their implant preference since the start of their practice, with “avoidance of secondary surgery” and “extrapolation from outcomes in adults” as the most cited reasons at 86.7% and 73.3%, respectively.

**Discussion:**

Our findings indicate that the shift from screw to suture-button fixation is due to an interest in avoiding secondary surgery for implant removal and by extrapolating results from adult studies.

Ankle injuries are common in the pediatric population, accounting for approximately 30% of pediatric sports medicine clinic visits.^1^ Injuries to the ankle syndesmosis, however, are far less common, accounting for less than 1% of pediatric patients presenting with ankle trauma.^2^ Also termed ‘high ankle sprains,’ syndesmosis injuries are defined as a disruption to the supportive ligaments between the tibia and fibula and are found to occur most frequently during sports that involve cutting and pivoting movements such as football,^2^ soccer, skiing, and hockey.^3^ When these injuries are associated with instability of the ankle mortise (either in association with a fibula fracture or not), it has been shown that fixation yields improved short-term and long-term functional outcomes as well as decreased pain in adult cohort studies.^4^ Historically, surgical syndesmotic ankle injuries were treated almost exclusively with static screw fixation. Since its release in 2008, however, suture-button fixation has grown in popularity, with multiple systematic reviews and meta-analyses in the adult population demonstrating superior results in functional outcomes,^5-11^ complication rates,^5,7,8^ implant failure rates,^5,8,9,11,12^ revision surgery rates,^6,8,10,11,13^ and time to weight-bearing.^7,11,12^ Similar data comparing outcomes between static and dynamic fixation methods for syndesmotic injuries are lacking in the pediatric population. Despite the lack of evidence, we have noted at our own institution that management of syndesmotic injuries in pediatric and adolescent patients has shifted toward increased use of suture-button fixation over time. The purpose of this study was to investigate trends in syndesmotic injury management by conducting a pilot survey of pediatric orthopaedic surgeons fellowship-trained in pediatric orthopaedic trauma. This study aimed to examine trends in pediatric syndesmotic injury treatment over the past 10 years and identify the motives driving these treatment preferences.

## Methods

At the time of data collection for this study, there was no previously validated survey available to assess surgeon preferences in the surgical management of pediatric syndesmotic injuries. Therefore, the investigative team developed a series of survey questions designed to gather information on the current surgical preferences of pediatric orthopaedic surgeons regarding the management of syndesmotic injuries and the factors influencing these preferences. The survey data were stored and managed within Research Electronic Data Capture.^14,15^

The opening survey question served to screen out any surgeons who did not surgically treat pediatric syndesmotic injuries at least once annually. Survey questions used branching logic, and respondents not meeting these baseline inclusion criteria were immediately directed to the ‘end’ of the survey. The remaining questions pertained to surgeon demographics, surgeon-reported implant preferences for surgical pediatric syndesmotic injuries, changes in management over time, and factors contributing to screw versus suture-button implant preference. The survey also asked surgeons to describe the aspect of pediatric syndesmotic injury management they found to be the most challenging. Most of the questions were close-ended multiple-choice queries permitting a single response. Questions pertaining to rationale for decision making included ‘Other,’ followed by an open-response comment field, as one of the multiple-choice answers. The survey was designed with assistance from a senior biostatistician to maximize questionnaire validity and optimize completion rates.

Cognitive validity was completed with three pediatric orthopaedic surgeons at our own institution. Revisions to the survey instrument included changes to the question sequence and survey design, rephrasing of questions for clarity, and grammatical changes. The survey was then distributed on May 12, 2020, to all members of the Children's Orthopaedic Trauma and Infection Consortium for Evidence-Based Studies (CORTICES, www.cortices.org), an organization comprising 30 pediatric orthopaedic surgeons from 20 different academic medical centers across the United States dedicated to the improvement of pediatric orthopaedic surgical care. For the purposes of this study, it was ensured that the following criteria were met by all the CORTICES respondents:

- Completed fellowship training in the program for pediatric orthopaedic surgery
- Still practicing as an operating surgeon in the United States
- Performed at least one repair of pediatric syndesmotic injury surgically per year, using either a screw or suture button
- Does not have any personal financial/contractual obligations to implant-associated entities that affect implant preference/choice for surgical repair.

Survey recipients were to complete the survey by May 21, 2020, and were sent two reminders to complete the survey before the deadline.

## Statistical Analysis

Data were groomed and validated for out-of-range and incomplete responses. The nonparametric signed-rank test was used to assess for significance of differences in continuous variables. The small sample sizes did not allow for extensive statistical analysis. Frequency and contingency tables were visually assessed. Where allowed, the Fisher exact test was used to assess for statistically significant differences. All tests were 2-sided, and a *P*-value <0.05 was considered statistically significant. SAS (version 9.4, SAS Institute) software was used to complete all analyses.

## Results

**Figure 1:**
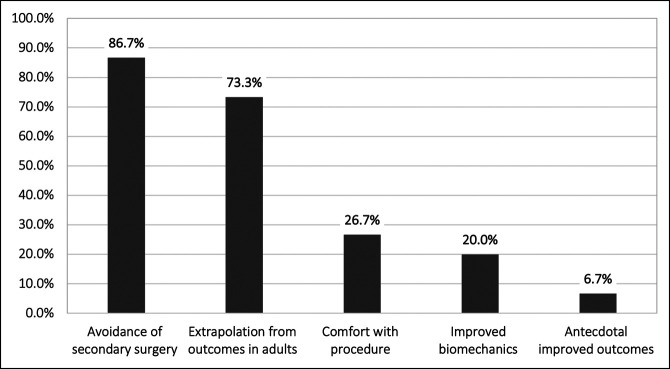
Bar graph demonstrating surgeon rationale for change in treatment choice, denoting stated reasons for changing the method of treatment for syndesmotic ankle injury.

All 30 recipients completed the survey, equating to a 100% response rate. A preference for suture buttons was reported in 70% (n = 21) of respondents, versus 30% (n = 9) indicating a preference for screw implant when surgically managing syndesmotic injuries in pediatric patients. Of those indicating screw preference, 66.7% (n = 6) reported routinely removing the screw prophylactically, with all six indicating a time interval preference of waiting 3 to 6 months after primary operation to do a secondary operation for screw removal. Exactly 50% (n = 15) of total respondents reported a change in implant preference since the start of their practice. The 15 surgeons, reporting a change in preference, cited the following reasons for their change in practice: ‘avoidance of secondary surgery,’ ‘extrapolation from outcomes in adults,’ ‘comfort with procedure,’ ‘improved biomechanics,’ and ‘anecdotally improved outcomes’ for 13 (86.7%), 11 (73.3%), 4 (26.7%), 3 (20.0%), and 1 (6.7%) of the respondents, respectively (Figure 1).

**Figure 2:**
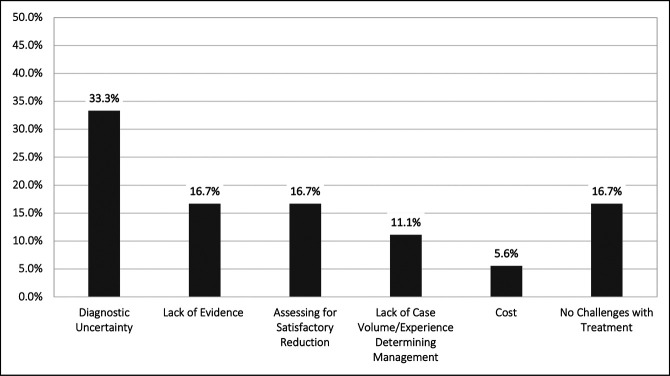
Bar graph demonstrating the greatest challenge for management of pediatric syndesmotic ankle injuries, denoting stated greatest challenges for treatment of syndesmotic ankle injury in the pediatric population.

Furthermore, respondents were asked to cite their greatest challenge associated with effectively managing syndesmotic ankle injuries in the pediatric population. Of the survey respondents, 18 (60.0%) answered the survey question. Diagnostic uncertainty, lack of evidence to guide management, and assessing for satisfactory reduction were the three most common responses with 6 (33.3%), 3 (16.7%), and 3 (16.7%), respectively (Figure 2).

Surgeons' years in practice varied; 26.7% (n = 8) of respondents reported less than 5 years, 53.3% (n = 16) reported 5 to 10 years, 13.3% (n = 4) reported 11 to 15, and 6.7% (n = 2) reported >15 years in practice as an attending. Of the respondents, 86.7% (n = 26) reported surgically treating 0 to 5 pediatric syndesmotic injuries in the past year, 10% (n = 3) responded that they treated between 6 and 10 of these injuries in the past year, and one respondent (3.33%) reported treating over 10 pediatric syndesmotic injuries surgically in the past year. Among all of those surveyed, 86.7% (n = 26) reported practicing in a metro setting (defined as a population greater than 190,000 people), 10% (n = 3) reported practicing in an urban/rural setting (defined as a population between 40,001 and 190,000 people), and one surgeon did not respond to the question (3.33%). The majority of surveyed surgeons (73.3%, n = 22) reported working in a pediatric specialty hospital, with the remaining 26.6% (n = 8) indicating they work in a tertiary care hospital (adult and/or pediatric hospital) setting (Table 1). Overall, survey responses showed a trend toward using suture buttons for syndesmotic injuries in pediatric patients after introduction of the implant to the market in 2008.

**Table 1:** Respondent Characteristics

Characteristic	Complete Cohort (n = 30) 100%	Implant Preference	
		Suture Button (n = 21) 70%	Screw (n = 9) 30%
	N (%)	N (%)	N (%)
Years in practice			
<5	8 (26.7%)	7 (33.3%)	1 (11.1%)
5-10	16 (53.3%)	11 (52.4%)	5 (55.6%)
11-15	4 (13.3%)	2 (9.5%)	2 (22.2%)
>15	2 (6.7%)	1 (4.8%)	1 (11.1%)
Practice setting^a^	n = 29	n = 20	n = 9
Metro	26 (89.7%)	18 (90.0%)	8 (88.9%)
Urban	3 (10.3%)	2 (10.0%)	1 (11.1%)
Type of hospital			
Pediatric hospital	22 (73.3%)	17 (81.0%)	5 (55.6%)
Tertiary care hospital	8 (26.7%)	4 (19.0%)	4 (44.4%)
Pediatric syndesmotic injuries treated *(per year)*			
<5	26 (86.7%)	18 (85.7%)	8 (88.9%)
5 to 10	3 (10.0%)	2 (9.5%)	1 (11.1%)
>10	1 (3.3%)	1 (4.8%)	0 (0.0%)
Changes in implant practice *(since start of practice)*			
Yes	15 (50.0%)	15 (71.4%)	0 (0.0%)
No	15 (50.0%)	6 (28.6%)	9 (100.0%)

a Differing n's are due to missing response data.

## Discussion

This study was designed to identify trends in surgical management of pediatric syndesmotic injuries at the nationwide level over the past 10 years, as well as the factors that may be influencing these trends. Our results suggest confirmation of the clinical observation of the principal investigators for this study, i.e., the implant preference in the context of pediatric syndesmotic ankle injury repair has changed over the past 10 years. Exactly half of the respondents indicated a change in implant preference since the start of their practice. Interestingly, 100% of the changes in implant preference were from screws to suture buttons.

Our results suggest that the reasons driving management preferences are multifactorial; 11 of the 15 surgeons (73.3%) reporting a change in preference from screws to suture buttons selected more than one option on the survey question inquiring about factors contributing to this change. Because the question had only six response options to choose from, it is likely that there are additional contributing factors not captured within the scope of this survey.

The widely accepted method of management for syndesmotic ankle injuries continues to evolve, and this study suggests a shift in practice among 30 pediatric orthopaedic surgeons that parallels what has occurred in adult patients over the past 10 years.^16-19^ Despite a growing body of literature comparing adult outcomes achieved with screw versus suture-button implants, limited evidence exists regarding pediatric patient management. Current literature studying adult patients demonstrates similar-to-superior short-term and long-term outcomes with suture-button versus screw utilization.^20-26^ In addition, while the individual cost of the suture button has been estimated to be as high as 10 times the cost of the syndesmotic screw, the overall cost of suture-button treatment may be cheaper when accounting for high rates of implant removal with syndesmotic screw treatment.^27^ The lack of consensus observed among respondents in this study reflects the dearth of evidence-based recommendations in the literature for the pediatric population.

There are several limitations to this study. The surveyed sample size was small (n = 30) because this was designed to be a pilot study. Of those surveyed, most of the pediatric orthopaedic surgeons (24/30, 80%) had 10 or less years of practice, which can suggest that the findings in the study are more representative of treatment practices of younger surgeons. However, the fact that all 15 surgeons who switched treatment methods changed from screws to suture buttons including three surgeons with over 10 years of experience indicates that the trend toward use of suture buttons for treatment of ankle syndesmotic injury in the pediatric population is not limited to younger surgeons. Although CORTICES members represent a geographically diverse set of institutions, it must be noted that most survey respondents in this study are associated with either pediatric or tertiary hospitals capable of providing specialized care. The resources available at these institutions likely influence choices in management, and the responses from the CORTICES group may not be generalizable to the practices of pediatric orthopaedic surgeons at all types of institutions. Their responses also reflect their own practices and may not be a comprehensive representation of the practices of all pediatric surgeons at their respective institutions. This study will inform a future large-scale national survey aimed at documenting treatment strategies for syndesmotic management by pediatric orthopaedic surgeons. In addition, all the surgeons surveyed were from the United States, which may not represent the practice norms internationally. Furthermore, this survey may have been affected by recall bias; surgeons were asked to indicate their preferences in management over the past 10 years, which may be skewed by their unique individual experiences during that time. Finally, there are likely additional factors contributing to surgeon practice preferences that were beyond the scope of our survey questions.

The results of this study are based on survey data and intended to provide a commentary on current management trends and preferences rather than to formulate recommendations on optimal management. Our survey data suggest that several surgeons prefer a dynamic (suture-button) implant to static screw fixation, and that several surgeons' implant preference has changed from screws to suture buttons over time. The rationale for this change in preference are centered on surgeons' wishing to avoid secondary surgery and the idea that superiority of suture-button implants can be extrapolated from research conducted with adult patients.^5^ This study serves as a starting point in recognizing that there has been a shift in management trends for pediatric syndesmotic injuries and strongly suggests that there is a clear need in future high-quality prospective studies to evaluate surgical approaches to better define the standard of care. The primary limitation of this study is our small sample size. Therefore, surveying a larger group of respondents is necessary to provide a comprehensive understanding of nationwide surgical trends in implant preferences.

Over the past 10 years, suture-button implants have gained popularity as an alternative to screw fixation for pediatric and adolescent syndesmotic ankle injuries. This information serves to identify the current trends in pediatric syndesmotic injury management and provides guidance for future investigations that aim to establish evidence-based standard of care for these challenging injuries.
